# Experiences of crisis care among service users with complex emotional needs or a diagnosis of 'personality disorder', and other stakeholders: systematic review and meta-synthesis of the qualitative literature

**DOI:** 10.1192/bjo.2022.1

**Published:** 2022-02-24

**Authors:** Kristiana DeLeo, Lucy Maconick, Rose McCabe, Eva Broeckelmann, Luke Sheridan Rains, Sarah Rowe, Sonia Johnson

**Affiliations:** Division of Psychiatry, University College London, UK; Division of Psychiatry, University College London, UK; and Camden and Islington NHS Foundation Trust, UK; School of Health Sciences, City University of London, UK; Health Service and Population Research Department, NIHR Mental Health Policy Research Unit Complex Emotional Needs Lived Experience Working Group, Institute of Psychiatry, Psychology & Neuroscience, King's College London, UK; Division of Psychiatry, University College London, UK; Division of Psychiatry, University College London, UK; Division of Psychiatry, University College London, UK; and Camden and Islington NHS Foundation Trust, UK

**Keywords:** Borderline personality disorder, self-harm, personality disorders, qualitative research, crisis intervention

## Abstract

**Background:**

Mental health crises are common in people with complex emotional needs (our preferred working term for people diagnosed with a 'personality disorder'), yet this population is often dissatisfied with the crisis care they receive. Exploring their experiences and views on what could be improved, and those of carers and healthcare staff, is key to developing better services.

**Aims:**

We aimed to synthesise the relevant qualitative literature.

**Method:**

Five databases were searched. Eligible studies included service users with a diagnosis of personality disorder and their carers or relevant staff, focused on crisis responses and used a qualitative design. Data were analysed with thematic synthesis.

**Results:**

Eleven studies were included, most focusing on emergency departments. Four meta-themes emerged: (a) acceptance and rejection when presenting to crisis care: limited options and lack of involvement of carers; (b) interpersonal processes: importance of the therapeutic relationship and establishing a framework for treatment; (c) managing recovery from a crisis: clear recovery plan and negotiating collaboration; and (d) equipping and supporting staff: training and emotional support.

**Conclusions:**

Our findings suggest that emergency departments have major limitations as settings to provide crisis care for people with complex emotional needs, but there is a lack of research exploring alternatives. The quality of the therapeutic relationship was central to how care was experienced, with collaborative and optimistic staff highly valued. Staff reported feeling poorly supported in responding to the needs of this population. Research looking at experiences of a range of care options and how to improve these is needed.

People whose difficulties fit the criteria for one or more personality disorder diagnoses make up a large proportion of people using mental health services, with up to 52% of people accessing out-patient services and 70% of in-patient and forensic patients in the UK reported to meet these criteria.^1^ We are aware of important critiques of the term ‘personality disorder’ because of its long association with stigma and therapeutic pessimism: we therefore prefer the working term ‘complex emotional needs’ (CEN) to describe people who may receive this diagnosis.^2^ We advocate co-produced and theoretically driven work to develop a valid and acceptable conceptual framework and terminology.

There are significant public health and individual well-being implications associated with CEN, including extensive use of healthcare resources, elevated suicide rates and reduced life expectancy.^1^ Mental health crises are a common experience among people living with CEN.^3^ A mental health crisis is when someone needs urgent or emergency help for their mental health; in people with CEN, this can involve an experience of acute distress, reduced ability to function and self-harm.^4,5^ The risk of a service user with CEN engaging in self-harm and other harmful behaviour is high in a crisis, especially as these behaviours can be used as coping mechanisms to regain control and manage symptoms of dissociation (a feeling of being disconnected from yourself or the world around you) or dysregulation (difficulty in managing emotional responses).^6–8^ Perceptual distortions can also occur in a crisis, and can lead to an increased risk of suicide plans and attempts.^7^

The goal of crisis intervention is to restore functioning and allow personal and social resources to be mobilised without a loss of control or negative outcomes.^9,10^ Crisis care takes place in a range of contexts, from hospital settings like emergency departments or brief admission wards, to models such as crisis houses or crisis teams that are intended as alternatives to in-patient admission.^7^ Little is known about the most helpful aspects of crisis care for service users with CEN.^11^ Previous research suggests service users with a diagnosis of a 'personality disorder' are generally dissatisfied with the crisis care they receive.^7,12,13^

## Existing literature

Previous reviews have synthesised qualitative research on treatment for people with a diagnosis of personality disorder;^2^ however, these focus on long-term out-patient and community care. Few studies focus specifically on crisis care for people with CEN. The small number of studies examining this found that care needs to be easily accessible and person-centred, with an element of joint decision-making between service users and professionals. Care was also experienced as more helpful if clinicians prioritised the development of therapeutic relationships.^7^ Wood and Alsawy's^14^ qualitative review on experiences of psychiatric in-patient care across all mental health conditions found that for service users, collaborative care, positive relationships and a safe therapeutic environment were important. Warrender et al performed an integrative review of the quantitative and qualitative literature on crisis intervention for people diagnosed with borderline personality disorder up to 2017.^7^ This study included only people with borderline personality disorder, excluding other subtypes; excluded people with comorbidities; used mixed methods rather than exploring the qualitative data in depth, and further papers have been published in this area since 2017. Therefore, a more up-to-date and in-depth review is warranted. To date, no systematic review has examined service user, carer and staff perspectives on crisis care for people with CEN receive and been inclusive of all personality disorder diagnoses. Widespread improvements to services for people with CEN have been called for from bodies such as the Royal College of Psychiatrists^15^ and the Department of Health,^16^ which, if implemented, will necessitate understanding of good practice and the barriers to achieving this, from both the service user and practitioner perspective. Evidence on such perspectives is therefore of high current importance.

The aim of this review was to conduct a meta-synthesis of the qualitative literature on crisis care for people with CEN, focusing on an in-depth exploration of service user, carer and staff experiences of care. A secondary aim was to explore stakeholder views on good practice and what improvements could be made.

## Method

We undertook a synthesis of relevant qualitative papers, using a meta-synthesis approach.^17^ The method for this meta-synthesis is reported according to the Preferred Reporting Items for Systematic Reviews and Meta-Analyses (PRISMA) checklist.^18^ The protocol was pre-registered on Open Science Framework (osf.io/wjs4t). The initial study focus and design was discussed with a lived experience researcher, who has written the lived experience commentary on this paper.

Ethical approval was not required for this study as it was a systematic review of existing literature and no original data were used.

### Eligibility criteria

Published peer-reviewed studies were eligible for inclusion if they met the following criteria. First, at least 50% of participants in the study must have been given a diagnosis of personality disorder or refer to a term which they explain to be an alternative to the term personality disorder (for example, one study referred to ‘emotionally dysregulated clients’, whom they identified as having been given a personality disorder diagnosis). Studies that included people with all mental health diagnoses using crisis care were eligible if they reported results for a subgroup of people with a diagnosis of personality disorder and this subgroup was at least 50%. Second, they reported findings from adults aged 18 years or above. Third, the care received can be considered crisis care. Types of care considered crisis care could include teams providing intensive treatment at home in a crisis (sometimes called crisis teams or home treatment teams), crisis houses and care received in the emergency department. Services could be specific to personality disorders or generic healthcare services. Forensic settings were excluded, as well as any service not intended for people in crisis or an acute phase of illness. In hospitals, short-stay wards and clinical decision units (services providing short-term interventions aiming to remove people from emergency departments and to avoid admission to a general acute ward if possible) were eligible, whereas general in-patient wards, in-patient wards for rehabilitation or specialist long-term treatment for those diagnosed with a personality disorder were excluded, as in-patient care has been specifically reviewed elsewhere.^14^ Fourth, the study was of a qualitative design. Fifth, studies were written in English. Finally, studies were published since 2000 to ensure that findings are relevant to contemporary services.

### Search strategy and study selection

Studies were identified by conducting a literature search across five databases: Medline, EMBASE, PsycINFO, Social Policy and Practice Database and Health Management Information Consortium, accessed via the Ovid electronic database platform. Reference lists of eligible papers were screened for additional studies and forward and backward citation searching was performed. The search included all studies published since 2000 to ensure models of care were comparable with current practice. Included studies were limited to English. A scoping search and preliminary reading was performed from June 2020 with the final search and data extraction conducted from the 23 November 2020.

**Fig. 1:**
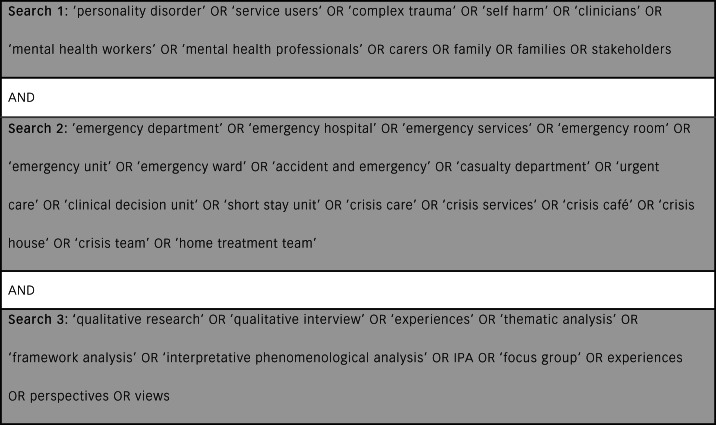
Literature search terms.

Search terms were built around keywords pertaining to stakeholders, crisis services and qualitative methodologies (see Fig. 1). Studies were first screened for relevance to the review topic by title by the first author. For abstracts of relevant studies or studies of unclear relevance, the full text was then screened. Fifty per cent of the abstracts were screened independently by two reviewers, to ensure agreement. Differences were discussed by the two reviewers until consensus was reached. If there continued to be disagreement, then this was discussed with a third senior author.

### Data extraction

Data on study characteristics were extracted, including study authors, year of publication, study setting, type of acute care, country, number of participants and participant characteristics, method of data collection and analysis of the quality of paper. Fifty per cent of the data extraction was checked by a second reviewer. The findings of included studies were exported to NVivo 12 (QSR International Pty Ltd. (2018); see https://www.qsrinternational.com/nvivo-qualitative-data-analysis-software/home) for inductive coding.

### Quality appraisal

All included papers were critically appraised with the Joanna Briggs Institute (JBI) tool.^19^ Quality appraisal was conducted independently by two reviewers and then discussed to resolve disagreements. Studies were not excluded on the basis of quality score; however, the quality of studies was considered when discussing strengths and limitations of the existing literature.

### Data synthesis and analysis

Thematic synthesis was conducted to analyse the data.^20^ Inductive line by line coding was carried out on NVivo and included all text presented in the ‘results’ and ‘discussion’ sections of included papers. Line-by-line codes were then grouped to start developing descriptive themes, taking into account similarities and differences. Iterative coding was conducted within the developing coding framework. We then developed more conceptual meta-themes and subthemes relevant to the review question. The first stage, line-by-line coding, was conducted independently by two reviewers, one of whom is a student researcher and one a clinical academic psychiatrist, who then collaborated on developing descriptive and meta-themes. The final coding framework was shared and further developed with collaborators who are experts by experience. Emerging themes were reviewed with the research team, which included a senior psychology academic with a specialist interest in communication in mental health settings, including emergency settings, a post-doctoral policy researcher, an academic psychologist who specialises in self-harm research, and an academic psychiatrist whose specialist interests include crisis care.

## Results

### Study inclusion and characteristics

**Fig. 2:**
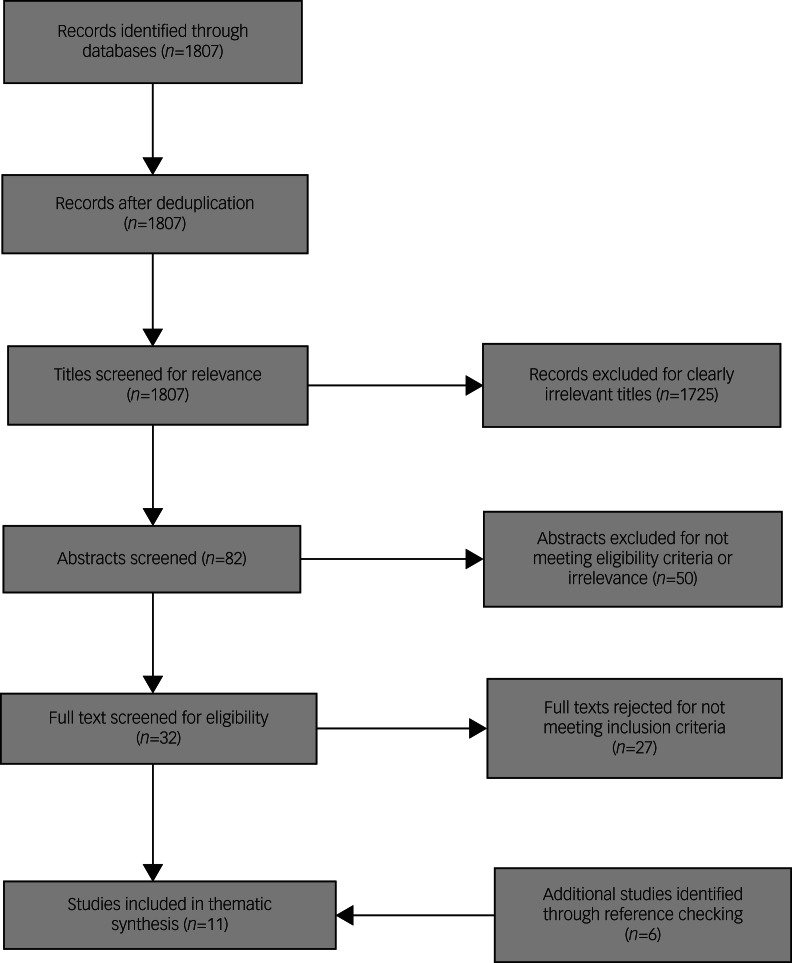
Preferred Reporting Items for Systematic Reviews and Meta-Analyses flow diagram.

Eleven studies were included (Fig. 2, Table 1). Studies were excluded at full-text stage because they used a quantitative design (*n* = 2); focused on crisis care, but did not report findings for people with diagnosis of personality disorder or CEN specifically (*n* = 20); or they reported findings for people with personality disorder diagnosis or CEN using general services, but not crisis care (*n* = 5).

**Table 1:** Summary of included studies

Study authors	Year of publication	Setting (type of acute care)	Country	Participant number/characteristics	Method of data collection	Date of data collection	Method of analysis
Dunne and Rogers^21^	2013	Community Personality Disorder Service covering a rural county in the East of England: one of two specialist personality disorder services in the region	UK	Eight carers of service users diagnosed with BPD. Five male carers and three female carers took part; relationship to the service user included four partners, three parents and one sibling	Two focus groups conducted, the first focusing on ‘the role of mental health services’ and the second focusing on ‘experiences in the community’. Focus groups were audio-recorded and transcribed	Unclear. Two focus groups; all eight carers attended the first, whereas five of the eight attended the second	Thematic analysis
Helleman et al^10^	2014	Out-patient participants with previous experience of brief admission asked to participate by their community clinician. All in care at a large mental health facility in a semi-urbanised, eastern region. Brief admission offered in four psychiatric clinics, patients are admitted for 1–3 nights	The Netherlands	17 service users with a diagnosis of BPD, Dutch-speaking with the ability to tolerate an interview	Qualitative, in depth interview with a 45–75 min duration. Interviews were guided by a memory aid and based on clinical experience/review of the relevant literature. The memory aid consisted of key words used with the research question to guide the participants	January 2011 to August 2012. Seventeen interviews were conducted, data saturation was reached after 15 interviews	Descriptive phenomenological methodology
Lohman et al^3^	2017	Data obtained from the Borderline Personality Disorder Resource Center at New York Presbyterian Hospital. The Center is an online centre connecting people with BPD and their families/friends to treatment and support	USA	Random subset of 500 transcripts participants who were aged >18 years, English-speaking and referred to services in the USA. Most inquiries were made by family/friends of people diagnosed with BPD, other inquiries were made by people diagnosed with BPD, as well as health professionals	Data was collected retrospectively from the Center's database of service requests, using telephone call transcripts	January 2008 to December 2015	Qualitative content analysis
Morris et al^22^	2014	Participants recruited through voluntary sector organisations in the North-West of England to discuss their experiences with general adult mental health services	UK	Nine service users diagnosed with BPD with a ‘significant’ period of contact with general adult mental health services in the past 3 years. Participants were aged 31–47 years, two males and seven females, eight described as White British and one as British. All participants reported comorbid Axis I and/or Axis II diagnoses	Semi-structured interviews including open and closed questions with a flexible interview schedule approved by a service user group. Interviews lasted 40–90 min, were audio-recorded and transcribed	Unclear	Inductive thematic analysis
Borschmann et al^9^	2014	Three community mental health teams in South London, all participants were community-dwelling adults who had created a crisis plan in a previous trial	UK	Forty-one adults diagnosed with BPD. A majority of these participants were White, female, single and in their 30s	One-time joint crisis plan meeting facilitated by a clinical psychologist, where an open discussion took place regarding aspects of the individual's crisis plan	January 2010 to May 2011. Forty-one one-time meetings were conducted	Thematic analysis
Spence et al^23^	2008	Emergency department at St. Michael's Hospital in Toronto	Canada	Twenty-five males aged 18–45 years, with a presenting complaint of suicidal ideation or any self-reported history of suicidal intent or a history of substance misuse problems. Seventy-five per cent of participants were diagnosed with BPD and 71% had an antisocial personality disorder diagnosis. Other personality disorders diagnosed in the sample included depressive, paranoid, avoidant, passive–aggressive, obsessive–compulsive, narcissistic and schizoid. Seventeen emergency department staff were interviewed (six registered nurses, five physicians, two crisis team workers, two security officers and two non-medical staff members)	Semi-structured and diagnostic interviews lasting 45 min-2 h for patients and semi-structured interviews lasting between 30 and 90 min for staff	January to October 2004. Twenty-five service user interviews and 17 staff interviews were conducted	Qualitative analysis using an iterative coding process
Burke et al^26^	2019	Public mental health setting, included a 7-h Clinician Connections workshop	Ireland	Thirteen emergency department and community mental health clinicians; 12 female and 1 male	Three focus groups conducted after completion of the Clinician Connections workshop	Over a period of 2 months	Thematic analysis
Vandyk et al^6^	2019	Emergency department at a university-affiliated tertiary care hospital in Eastern Ontario	Canada	Six service users with a primary diagnosis of BPD who frequently present to the emergency department	Semi-structured interview lasting 1 h	Spring and summer of 2016	Interpretive description
Barr et al^24^	2020	Consumer, carer support groups and community personality disorder services	Australia	Eight consumers and seven carers recruited by a flyer advert through consumer and carer support groups and services. Five out of eight consumers were female, six out of seven staff were female; mean age 36.75 years. No ethnicity recorded	Two focus groups: one with consumers and one with carers	On one day, unclear when	Reflexive thematic analysis
Eckerström et al^25^	2020	Brief admission to two wards in a psychiatric clinic in Stockholm, which specialise in the treatment of people with emotional instability and self-harm	Sweden	Fifteen participants recruited consecutively through out-patient unit; documented clinical history of emotional instability, history of self-harm and participant has enquired about brief admission at least once. 87% female.	Semi-structured interviews at out-patient units	October to November 2017	Thematic analysis
Commons Treloar^27^	2009	Emergency department	Australia and New Zealand	140 health practitioners; 64.3% general mental health services, 35.7% emergency department employees; 69.3% nurses, 17.1% allied health and 13.6% medical practitioners	Responses to open comment section of a questionnaire	Unclear	Thematic analysis

BPD, borderline personality disorder.

Of the included studies, three were conducted in the UK,^9,21,22^ two were from Canada,^6,23^ one study each was conducted in Australia,^24^ the USA,^3^ Sweden,^25^ Ireland,^26^ and The Netherlands,^10^ and one study was conducted across Australia and New Zealand.^27^ Sample sizes ranged from six to 500 participants (the study that used 500 participants performed content analysis^3^). The majority of papers (*n = 8*) used thematic analysis. Other analytic approaches included descriptive phenomenological methodology (*n =* 1),^10^ unspecified qualitative analysis (*n = 1*)^23^ and content analysis (*n* = 1).^3^

Study settings included emergency departments and international equivalents,^6,23,26,27^ brief admission units^10,25^ and a personality disorder resource center,^3^ which was a central point of contact in a USA district that connected service users to appropriate mental health services, including crisis services. There were no studies exploring experiences of crisis teams or other community options for crisis care, such as crisis cafes, acute day units or crisis houses. As a result, the findings are focused on experiences of accident and emergency (A&E) rooms and brief in-patient admissions. In this paper, brief admission refers to an intervention developed in The Netherlands specifically for people with a diagnosis of borderline personality disorder, and is different from general psychiatric hospital admissions.^28^ It is a maximum of three nights, the service user can self-refer and manages their use of the service, such as managing their own medication. The service user sees a nurse rather than a psychiatrist and makes use of a treatment plan agreed with the service user before admission.^10^ Populations studied included service users with a diagnosis of personality disorder and service users with comorbid substance misuse and a diagnosis of personality disorder. Only one paper included a service user population with confirmed diagnoses of personality disorder other than borderline personality disorder: this included participants with a diagnosis of antisocial personality disorder.^23^ One study looked at staff perspectives,^26^ which included A&E staff and community mental health clinicians. Two papers focused on family carers.^21,24^ Six papers provided information on the gender of participants,^6,9,21–23,26^ and only two reported ethnicity.^9,22^ Of the papers that did report ethnic background, the majority of participants identified as White.

Overall study quality was determined to be high, with the majority of papers showing congruence between the research question, methodology, methods of data collection and presentation of results. However, most papers failed to adequately describe the researchers’ philosophical and cultural position or influence on the research, a measure of quality on the JBI tool.

### Findings

#### Meta-theme 1: acceptance and rejection when presenting to crisis care

##### Subtheme 1.1: impact of limited care options and capacity

Whether service users felt accepted or rejected when presenting to crisis care was a salient part of their experience. Specialist crisis care for people with CEN was rarely described, and there were many descriptions of service users being referred to community services that they did not qualify for or that did not meet their needs.^3,22,23^ In a crisis, a loss of ability to understand or articulate feelings is common, further compounding the difficulty for service users in expressing complex and changing needs.^10,23^ Service users reported that they needed crisis services to be accessible to enable them to seek help before the crisis becomes very severe.^3,10,21,22^ When service users felt listened to and felt they were able to trust clinicians, conversations could be key to overcoming a crisis.^10^ During the brief admission intervention, early conversations with a nurse in which the nurse was active and the service user felt listened to helped to create a sense of safety and reduce service users’ levels of emotion.^10^

Crisis teams and other alternative care options were seen as valuable,^3^ yet A&E was perceived by family carers, professionals and service users as often the ‘only option’ or ‘last resort’ during a crisis.^23^ When community resources or alternative crisis services were inaccessible or unavailable, A&E became the only option for care.^3,6,21^ Service users said that they felt forced to use A&E when their community worker was away, when the crisis was too severe for community care or they were on a long waitlist for alternative care.^6^ Staff in a variety of settings felt that their own service environment was not suitable for this patient group, with both A&E staff and general practitioners reporting that people with CEN would be better served by a service other than their own.^23^ There was the perception among staff that there were inadequate resources for this group of people, or that current services were inadequate for them.^27^ Staff reported that could feel uncertain as to how to respond to very dysregulated service users, and that conflicts could arise among treating teams when there were different options for how to proceed with care.^27^

Although A&E often seemed to be the most readily accessible crisis care service, A&E staff reported facing tremendous difficulties managing CEN and caring for these service users. They described lacking the resources to deal with high levels of distress, as the A&E environment can be overburdened and chaotic.^3,23^ The behaviour of some people with CEN was perceived as sometimes challenging in A&E, and thought to be a reaction to long waits,^23^ limited freedom or poor treatment from staff.^10^ Comorbid substance misuse was also a challenge. A&E staff reported that they lacked confidence in caring for people with comorbid problems such as substance use, which can be common in service users with CEN (Table 2).^3,6^

**Table 2:** Summary of themes and subthemes, with illustrative quotes

Theme	Subtheme	Quotes
Acceptance and rejection when presenting to crisis care	Impact of limited care options and capacity	‘The hospital is always my last resort … I end up feeling worse … and the waiting … it's more nerve-wracking for me. …’ (A&E service user)^23^ ‘But the only problem is like when you have concurrent disorders, it's really hard for them to manage them both at the same time’ (A&E service user)^6^
	Carers experience rejection and lack of engagement from healthcare systems	‘I still think we need to be involved more than what we have…It's us that have to deal with it 7 days a week’ (Carer)^21^
Interpersonal processes and dynamics in crisis care	Setting and negotiating a framework for treatment	‘Discuss with a patient what the expectations of the brief admission are. . . . Put this on paper, individually. What to expect from the clinic. Let this be clear’ (Service user of brief admission)^10^ ‘In the beginning, I went helping others, you know, but then I stopped. Now I say, just go to the nurse, that's what they're here for. I am here for my own problems’ (Service user brief admission)^10^
	Developing and maintaining the therapeutic relationship	‘…Please treat me with respect and don't be rude to me; treat me as a person, not as a person with mental health problems’ (Service user completing a crisis plan)^4^ ‘If they connect with you and ask you what went wrong or what they can do for you; that is so nice. The nurse talked with me for 30 min; it was a revelation. It removes a rock from my heart. I melted and felt heard, and I told her stuff’ (Service user of brief admission)^10^
	Challenging interpersonal interactions exacerbate presenting problems	‘There's been times when I've felt like the lowliest person in the world…I'm so apologetic and I'm so embarrassed, you know?’(A&E service user)^6^ ‘I notice a big difference if I'm coming in for a medical reason than if I'm coming in for a psychiatric reason … it's almost like they're fed up with me’ (A&E service user)^23^
Managing recovery from a crisis	Transitioning with a clear and integrated recovery plan	‘… be better linked…who is their telephone contact for phone coaching-whether they are attending the group’ (Clinician)^26^ ‘Even when [the person I support] was sent home from hospital two times, she was never sent home with any- thing… Not unless you ask for it’ (Carer)^24^
	Negotiating collaboration and service user responsibility	‘It's probably the approach to me – that I am encountered in a different way. More like an adult individual, because you have so much. You have free permissions. You take care of your medicine. So, it does mean that you have a responsibility for yourself, and that has made me a more independent person’ (Service user using brief admission)^25^
Equipping and supporting healthcare staff to provide quality care	Training, knowledge and confidence	‘The sense of helplessness, getting swallowed up and becoming almost dysregulated yourself’ (Clinician)^25^ ‘I feel inadequate’ (Clinician)^28^ ‘It takes away the impatience and the lack of empathy you can sometimes have’ (Clinician)^26^
	Emotional support and boosting morale	‘Peer support was reassuring…that others experience these difficulties too’ (Clinician)^26^

A&E, Accident and Emergency.

Because of these issues with suitability and accessibility of services, service users reported that they often felt help-seeking was ‘futile’. Professionals echoed this view, saying that for service users with CEN there is a chronic and frustrating ‘back and forth’ cycle of repeated emergency department use.^6,23^

The degree to which service users found care to be accepting or rejecting appeared to vary according to the setting. In the papers that explored experiences of brief admission, service users reported that knowing that the brief admission was accessible to them if needed made them feel safer when in the community.^25^ In contrast, A&E, although accessible, was considered a ‘last resort’.^23^

##### Subtheme 1.2: carers experience rejection and lack of engagement from healthcare systems

Family carers reported that their support needs were not considered adequately, their requests went unanswered**,** their relative's diagnosis and needs tended to be poorly communicated to them,^3,21^ and professionals often refused or were reluctant to involve them in treatment.^21^ Carers sought out psychoeducation on their own and sometimes acknowledged that their understanding of CEN was poor, yet they felt they were expected to make important treatment decisions in the face of contradictory and inconsistent advice.^3^ When asked about the possible reasons behind their exclusion from care at the time of a crisis, carers felt that staff were unaware of the critical role they played in the service user's life.^21^ A collaborative relationship with professionals was reported to be preferable for carers, providing greater communication and guidance on how to manage crises.^21^ Carers also wanted staff to recognise them as individuals outside of the caring role, and be more considerate of how the caring role affects all aspects of their life.^21^

Carers faced many practical issues, including financial strain resulting from the impact of the caring role on their ability to work. Resources to support carers were seen as inadequate, and emotional support was felt to be lacking.^3,21^ Some carers described experiencing a ‘parallel crisis’ with their own distress when the service user was self-harming or attempting suicide.^21^ If carers experience these painful crises repeatedly with no support offered, the caring role can become so painful and overwhelming that they see no choice but to walk away.^3,21^

#### Meta-theme 2: interpersonal processes and dynamics in crisis care

##### Subtheme 2.1: developing and maintaining the therapeutic relationship

Clinician characteristics appeared to have a large influence on crisis care quality. There is a strong convergence among service users and carers about the interpersonal qualities they valued. Across the board, non-judgemental and validating support was the most helpful aspect of the therapeutic relationship for both service users and carers.^6^ Service users emphasised the importance of openness, friendliness and warmth when presenting to crisis care.^10,24^ Service users also wanted staff to be genuinely engaged and interested in them, to feel seen and heard, and be treated with dignity ‘like a person’ instead of a ‘patient’.^22,29^ Staff who consistently exhibited these characteristics were able to build a trusting relationship with the service user.^25^ Strategies such as telling jokes, making small talk and participating in informal conversation helped put service users at ease and in a better position to trust clinicians.^10^

Service users were most encouraged by staff who took a recovery-focused, optimistic and hopeful approach. Staff who were non-judgemental, normalising and gave service users a ‘chance to recover’ rather than emphasising the perceived chronic or ‘life-long’ nature of CEN or their diagnosis were highly valued.^9,22^ Service users also discussed the importance of being viewed as an individual by the staff rather than just another person ‘moving through the system’. Flexible and holistic care was lauded as the ideal by service users, but many reported that they received treatment that was overly standardised and did not take their individual needs or history into account.^26^ Staff recognised that interactions with service users, particularly when service users were very emotionally dysregulated, could bring up uncomfortable emotions in themselves, including a sense of frustration and helplessness.^26,27^ In some staff this resulted in reflection and a request for more training, and in some it resulted in the expression of stigmatising beliefs that people with the diagnosis of borderline personality disorder were wasting health service time.^27^ Staff that participated in a workshop considering clinician connections with service users reported that a greater awareness of their own internal states and the impact of their own emotions on the interaction was helpful in developing good therapeutic relationships.^26^

##### Subtheme 2.2: setting and negotiating a framework for treatment

Interpersonal relationships with carers and staff can either greatly relieve or greatly add to the service user's suffering in a crisis. Service users and staff both stressed the importance of negotiating a clear framework for care. For example, for people using a brief admission intervention, care was most helpful if information on how and when they could contact nurses were provided early on and communicated clearly to service users.^10^ Being consistent and non-judgemental, maintaining professional boundaries and communicating clearly were considered to be among the most important staff characteristics. Yet many service users and carers experienced poor communication from healthcare staff, which hindered the development of a trusting relationship.^3,22^

Although staff understood and endorsed needs for sensitive, clear and considerate communication, some staff reported finding it difficult to strike a balance between having boundaries and being empathetic.^26^ Some staff were concerned they would be reinforcing ‘bad behaviour’ from service users by being validating and empathetic.^26^ Boundaries between service users were even harder to enforce and were less defined. Some service users became overinvolved in the care of others and then felt responsible for conflict de-escalation, which was inappropriate to them as they were meant to be focusing on their own recovery.^10^

The negotiation of responsibility in a collaborative relationship appeared to vary by setting. During a brief admission, participants described benefit from being helped to take responsibility for their care,^25^ by avoiding losing control and coercion. This was in contrast to in A&E departments, where staff's perception that the person with CEN could take control of their illness could drive negative attitudes and frustration.^23^ Service users reported experiencing A&E as noisy, chaotic and stressful, and often felt unsafe.^23^ Many service users expressed a preference for access to alternative crisis spaces, such as cafes, respite homes or rehabilitation centres.^24^ Service users also expressed the need for more freedom and autonomy in their care, but this was rarely provided in A&E.^25^ As a means to help address this issue, some service users endorsed the development of joint crisis plans, which detailed how they wanted to be treated by staff in a crisis and helped them feel more in control and satisfied with their care.^9,10^

##### Subtheme 2.3: challenging interpersonal interactions and negative staff attitudes can exacerbate presenting problems

Service users reported many experiences of marginalisation, stigma, discrimination and negative attitudes among healthcare staff.^3,6^ Common experiences were staff becoming dismissive and reluctant to engage, or having their care withdrawn completely based on their diagnosis or when presenting with problems such as self-harm.^3,6,22^ Service users felt they had been deprioritised for treatment or ‘put in the back of the line’ once staff discovered their diagnosis. Service users who had been identified and labelled as a ‘frequent flyer’, or someone who often presents to A&E, reported particular experiences of discrimination.^23^

Although some A&E staff did feel sympathy toward patient distress, service user background heavily influenced this.^23^ There was an attitude among staff that patients with CEN were time-consuming^27^ and taking time away from patients with acute medical problems, and this was felt to be particularly frustrating if they had a history of repeat admissions.^6,23^ Other problematic attitudes from staff included a belief that service users with CEN are ‘just choosing to be difficult’,^6^ they are ‘manipulative’,^27^ ‘attention-seeking’ and ‘using the system’ in a way that it was not intended.^23^ Service users also reported issues of diagnostic overshadowing and feeling ‘defined’ by their diagnosis.^22^ Many service users reported apprehension about receiving a diagnosis because of stigma, feeling fearful that a permanent label would lead to a curtailing of freedoms or loss of control, leading them to conceal or minimise their presenting problems.^3^ Staff also reported that once a person was given the diagnosis of borderline personality disorder this could lead to a loss of ‘objectivity’ in staff assessments of the person.^27^

Negative experiences elevated emotional distress and reinforced low self-worth with service users. They described feelings of rejection, abandonment, shame, isolation, helplessness and guilt after presenting to crisis care, especially A&E.^6,10,26^ Interactions with staff did not have to be overtly hostile to be negative; feeling ignored, misunderstood or dismissed also worsened negative emotions in the service user.^10^ Service users were less inclined to seek help after a negative experience with crisis care to avoid feelings of shame.^23^ This is potentially dangerous as future suicide attempts may be more resolute to avoid readmission.^6^

#### Meta-theme 3: managing recovery from a crisis

##### Subtheme 3.1: transitioning with a clear recovery plan

Service users explained that having a preventative strategy in place and knowledge of effective crisis resources provided a sense of security and reassurance after discharge.^10,25^ Service users were helped by the knowledge that they could access care when needed, be provided with support to continue any intensive therapies, and could stop future crises from getting worse with a quicker return to baseline functioning in daily life.^10,25^ However, many service users reported that links to out-of-hospital support were limited, particularly if they were usually high-functioning before the crisis occurred.^6^ This lack of access to continuing support meant that these service users had difficulty continuing in their recovery, leaving them more vulnerable to further mental health crises.^6^

Carers reported that discharge from emergency services was often sudden, confusing and unexplained.^24^ Often the service user was sent home without a clear treatment plan or appropriate support in place.^24^ Thus, carers felt their involvement in treatment was limited, as the plans for discharge were often not communicated to them despite their importance to recovery.^3,24^

##### Subtheme 3.2: Negotiating collaboration and service user responsibility

Service users wanted opportunities to self-refer for support and maintain some autonomy when in crisis; for example, being able to self-refer to a safe place when feeling suicidal, where hospital admission or police involvement would be unlikely to ensue.^6^ Despite these desires to take more responsibility for their care, service users often experienced a lack of autonomy, particularly in A&E and in-patient settings.^25^ They described experiences of constant supervision and coercive measures, which undermined their ability to take responsibility for their own treatment and recovery.^25^ More novel approaches, such as self-referral for brief admission, may help combat this, as service users who had experienced both brief admissions and regular admissions reported that they were encouraged to take an increased amount of responsibility during the brief admission.^25^ These service users explained that they embraced and appreciated the requirement to be more involved in their care planning.^25^ Advanced crisis plans were identified as one way that people with CEN can have more control over their own care during a crisis, in which participants coproduced crisis plans during periods of stability.^9^ The importance of individualisation of care was highlighted, as participants showed a wide range of different preferences, and the majority took the opportunity to include refusals for specific treatments, such as medication and involuntary detention.^9^

#### Meta-theme 4: equipping and supporting healthcare staff to provide quality care

##### Subtheme 4.1: training, knowledge and confidence

Clinicians at all levels of experience across services (community mental health teams, A&E, brief admission) reported lacking confidence in treating CEN, expressing that they often felt ‘helpless’ and ‘incompetent’ interacting with service users presenting with emotional dysregulation.^26,27^ Treating psychological distress successfully was a major area of concern, as clinicians felt their knowledge and skills were lacking in supporting service users during emotional crises.^26^ Clinicians reflected that uncomfortable emotions could be brought up by interacting with very dysregulated service users, and staff could often feel unsure of how to respond.^27^ This lack of confidence and understanding often led to frustration among healthcare staff, as they did not know why these service users expressed intense emotions and challenging behaviour.^26^

Service users and carers thought professionals needed to be more educated about CEN, as knowledge was seen as a central influence on staff attitudes toward people with a diagnosis of personality disorder.^6,21^ Misunderstandings could lead to negative beliefs and attitudes, which is damaging to service users seeking help. In contrast, further training around CEN helped change staff perceptions of service users, so that they were able to take a more positive view of their interactions.^26^ Professionals agreed that there was a major need for further training and opportunities to practice necessary skills for working with these populations, and most were keen to take part in this.^26^

##### Sub-theme 4.2: emotional support and boosting morale

Many professionals discussed difficulty in staying calm and regulating their own emotions when working with people in crisis. Burnout and high work stress were reported as significant risks when working with service users with CEN.^26^ This was exacerbated if staff did not have the proper skills and training to know how to support these service users in crisis situations.^26^ This is especially true for A&E staff, who often work in an already overburdened environment with competing demands on their time that may preclude the opportunity for support.^23^ Staff agreed that increased communication, good team support from peers at work and clinical supervision^27^ would help ease their workload burden, improve patient care and increase their confidence, whereas poor and unsupportive workplace cultures contribute to burnout and poor practice.^26^

## Discussion

Overall, most stakeholders did not have a positive experience of receiving or providing crisis care in crises related to CEN. A lack of accessible community and alternative crisis resources for service users were reported, often leading to A&E presentations, where they were more likely to be met with stigma, discrimination and negative attitudes. There was a high level of agreement between papers that when care was experienced positively, the quality of relationships and interactions between staff and service users were key. Staff could cultivate positive relationships by being genuinely interested in the service user, treating the service user as an individual and taking an optimistic stance toward recovery. The importance of recovery-oriented care, the value of therapeutic relationships and the need for easily accessible crisis care are consistent with prior research into non-crisis services for people with CEN.^2,30,31^

It appeared that when met with rejection and negative judgements, this could sometimes result in service users’ presenting problems being exacerbated by contact with health services. Risks to the service user were high in these situations, as service users reported they were likely to try to avoid crisis care in the future, so that risk of presentations occurring late, when distress is severe, may be exacerbated. Consistent with previous literature, discriminatory experiences could reinforce poor self-worth and become a repetition of previous life experiences.^30^ Feelings of shame when exposed to negative attitudes from staff had the potential to fuel another mental health crisis after discharge, contributing to the cyclical nature of crisis and admission for service users with CEN. In contrast, professionals who were seen as hopeful were valued, consistent with other studies that looked at experiences of wider mental health services for this service user group.^30^ Carers also felt excluded and rejected from healthcare systems. Carers described a sense of ‘all or nothing’ responsibility.^7^ Carers felt that they were expected to take responsibility and make treatment decisions in relation to a diagnosis they did not understand, yet they were not adequately involved by healthcare staff in the service user's immediate care during a crisis or beyond. They also reported a major lack of emotional and practical support, such as help with finances or access to support groups. This is consistent with previous research on the need for greater involvement of carers during mental health emergencies and joint decision-making for recovery.^32^

This review has addressed a different research gap from Warrender et al's previous review.^7^ This review has a greater focus on the setting for delivering crisis care, as the search strategy for this review specifically included the models of crisis care currently in use in the UK and some other countries, such as crisis teams and crisis houses, which were not included in the Warrender et al literature search terms.^7^ This approach has highlighted that there is no published qualitative research exploring service users’ experiences of these models. Additionally, the Warrender et al review^7^ considered only those with a borderline personality disorder diagnosis, whereas this review sought to include the literature for all personality disorder diagnosis subtypes. One study was found that reported findings for men with an antisocial personality disorder diagnosis attending the emergency department, which reported more experiences of becoming ‘disruptive’ or agitated in the emergency department and ‘losing control’.^23^

There is evidence from previous research on crisis care for other mental health difficulties that alternatives to hospital admission, such as crisis houses or home treatment teams, may provide a more positive experience for users of mental health services.^33,34^ One study found that the atmosphere in crisis houses is experienced among service users with varying mental health diagnoses as more calming, therapeutic and respectful than in-patient wards, when service users were given a greater degree of freedom and trusted more by the staff.^33^ Collaboration and mutual engagement, rather than coercion, is highly valued by crisis house staff and service users alike,^7,33^ and the importance of these features of a positive therapeutic relationship for people with CEN was identified in this review. These services can be accessed directly without requiring an attendance at A&E, potentially mitigating one area of negative experience for service users. In the UK, as part of the response to the COVID-19 pandemic, crisis assessment services have been developed separate from A&E departments,^35^ which may prove to be more acceptable environments for providing crisis care for people with CEN. Crisis teams are also generally quick to respond during a mental health crisis; therefore, referral to these kind of teams may address the issues of health service capacity and accessibility.^36^ Home crisis management as opposed to care in A&E or hospital was found to be preferable to many people with CEN creating advanced crisis plans.^9^ There has been some debate about whether crisis teams are a suitable model to work with people with a personality disorder diagnosis,^4^ but this has not yet been investigated in the research literature. Some less-intensive self-referral alternatives, such as crisis cafes, are being implemented in the UK and offer potentially useful models in which service users can access support before a crisis has escalated. However, whether alternatives such as crisis teams, crisis houses or crisis cafes provide an effective or acceptable form of care for people with CEN has not yet been specifically studied.

Staff recommendations for crisis care improvement focused more on staff well-being and containment of the emotional responses in staff that may arise when caring for people in distress.^27,33,36^ The concept of ‘containment’ from the psychotherapeutic literature^37^ helps to consider how the emotions of the service user are processed by the person aiming to provide care, and is key to staff members’ ability to respond in a helpful way to distress.^31,38^ In this review, staff reported an insufficient level of emotional support in the workplace, which is consistent with previous qualitative research on healthcare staff working with service users with CEN.^27^ In literature studying care for people with CEN across the mental healthcare system, staff reported difficult feelings being elicited in the course of their work, such as feelings of inadequacy or frustration, and being unsure how to respond to this.^27^ Previous findings indicate that staff need to have opportunities to process the emotional impact of their work,^31^ to avoid work-related burnout and maintain warmth and empathy, but this kind of support is not always provided by their workplace.^33^ There was an increased risk reported of feeling ‘burnt out’ among staff who work with service users with CEN, which can make staff less able or willing to actively make changes to fit service user and carer needs.

The findings of this study are consistent with a wider body of work conducted by the Mental Health Policy Research Unit (MHPRU) reviewing the qualitative literature of service user and clinician experiences of non-crisis community services for people with CEN.^2,39^ In longer-term community services, people with a personality disorder diagnosis can also face exclusion. Similar to crisis care, the provision of care was found to be often based on a subjective judgement of their need or motivation to engage. Services were found to be confusing and difficult to navigate, with service users having to advocate for themselves to access the care they needed. This suggests that many of the difficult interactions between providers and service users described in this review may not be unique to crisis care. However, in the MHPRU review, service users commented that negative experiences can be more common in general mental health services, and relatively less common in services specialised in looking after people with a personality disorder diagnosis.^2^ At present, there are very few crisis services designed with people with CEN specifically in mind. A common theme across both reviews is the centrality of the therapeutic relationship, highlighting the importance of services supporting and empowering their staff to be able to provide good care.

### Limitations

This review has several limitations. The research in this area is sparse, and therefore limits what can be said about specific crisis services. Given the small number of papers, it was also not possible to consider differences between the experience of healthcare systems in different countries, as no studies looked at comparable models in more than one country for the same group (service user or provider). Some clearly irrelevant papers were excluded at the title stage for speed; however, this approach means that there may be some papers that could have been missed where the title was unclear. There was particularly limited research on alternatives to hospital, such as crisis houses and crisis cafes – an important gap – and so the findings are focused on experiences of A&E and of brief admission to hospital. Only one paper included a confirmed diagnoses of a personality disorder other than borderline personality disorder, which was antisocial personality disorder. This is unsurprising, given that the emotionally unstable personality disorder or borderline personality disorder diagnosis predominates in mental health services, although it limits our ability to generalise the experiences described to all diagnoses.

The experiences also cannot be generalised to diverse countries and ethnic groups. The studies included in the review came from higher-income countries such as the UK, Canada, Sweden and Australia. No papers were rejected on the basis of language, as no papers found were in a language other than English. However, there may be studies not included in the databases searched or in the grey literature in other languages that were not captured by the search strategy. Many papers did not report on the ethnicity of participants, and of those that did, most participants were White. The majority of studies did not adequately describe the researchers’ philosophical or cultural standpoint or their own influence on the research, a measure of quality on the JBI assessment tool. This is a limitation as the interpretation of qualitative data will be affected by the position of the researchers, and the majority of papers were likely written from a clinician or academic perspective. These limitations highlight some important directions for future research.

### Directions for future research

Future research should focus specifically on the experiences and needs of people with CEN presenting in crisis, rather than general mental health populations, as crisis care in its current form appears to be in need of improvement to meet the needs of this patient group. Research looking at experiences of a wider range of available options for crisis care, including crisis teams, crisis houses, crisis cafes and other models, is also urgently needed. Because of the lack of research in this area, there is a need to expand on the diversity of populations with CEN studied, including a wider range of ethnicities and nationalities. It is not currently clear how services might be improved to be more suitable and accessible.

### Implications

There are significant barriers to providing good psychological care in the A&E setting, and staff reported that they feel they lack the training required to adequately support people with CEN. Alternatives to emergency departments that accept walk-ins or self-referrals with a relatively low threshold and a less medical approach, such as crisis cafes, crisis assessment units and crisis houses, may provide better experiences for service users, as the environment may be more therapeutic and service users may be able to take more responsibility for their own recovery. It is still unclear, however, which models of care provide the most positive experiences and better outcomes for service users.

Because of the importance of shared decision-making in care and recovery, as well as preserving service user autonomy, strategies such as joint crisis plans could be used more often. This could be a helpful strategy as it involves carers in the process and allows the service user to take a proactive and preventative approach, as some struggle to seek help during a crisis.^11^ When planning crisis services, managers and clinicians should design models that facilitate staff to build collaborative and trusting relationships between providers and service users. Brief admission interventions described in some of the included studies have been piloted in Holland^11^ and Sweden,^23^ and propose an alternative model to traditional in-patient admissions, in which service users are able to self-refer for brief admissions to hospitals, during which a greater level of autonomy and responsibility in the therapeutic relationship is placed in the hands of the service user compared with in a conventional admission.

More mental health training and emotional support in the workplace for staff is necessary to improve attitudes, quality of care and reduce burnout. Enabling staff to do their jobs with more confidence, knowledge and better mental health would ultimately result in better care for service users.

### Lived experience commentary by Eva Broeckelmann

‘Given my lived experience of the Serenity Integrated Mentoring (SIM) programme,^40^ which threatens to criminalise people with CEN in distress and routinely denies them access to essential crisis services,^41^ I am acutely aware of the importance of this topic. There are significant parallels between the dehumanising and coercive approaches of SIM in England and service users’ experiences of discrimination and lack of autonomy described in the international literature, which highlights the immense burden that the stigma of a personality disorder label places on service users around the world.

The fact that the evidence base on crisis services for people with CEN is so sparse speaks volumes and serves as stark reminder of the lack of parity between ‘personality disorders’ and other mental health conditions, which are generally considered more deserving of help in crisis. It is yet another area where our needs are not taken seriously, and this stigmatising diagnosis still too often leads to exclusion from care.

As documented in this meta-synthesis, A&E departments are undoubtedly one of the least suitable environments in which to access crisis care. Irrespective of the setting, however, hardly anything could be more invalidating and counterproductive than cold rejection from the very professionals whose job it should be to provide care and support at times when service users are at their most vulnerable. Especially for people with CEN who have often survived extensive, complex trauma, such experiences are nothing but re-traumatising and can be detrimental to recovery.

From a service user perspective, it can indeed be very noticeable when staff lack confidence treating people with CEN in crisis, but despite these professionals’ well-intentioned appeal for further training, I cannot help but wonder if their priorities are really congruent with service users’ needs. After all, effective crisis care for CEN is not about advanced specialist skills or knowledge, but relies primarily on very basic human qualities. What often matters most to service users is the intangible quality of the therapeutic relationship, with non-judgemental, empathetic staff who are emotionally available to see the person behind the label and respect them unconditionally.

Better co-produced and value-based training could certainly help to improve staff attitudes toward people with CEN. However, given the largely negative experiences with A&E departments, it is also imperative that more research and resources are invested into less restrictive, more accessible, individualised and holistic alternatives for crisis care beyond the medical model.’

## Data Availability

Data availability is not applicable to this article as no new data were created or analysed in this study.

## References

[1] Dale O, Sethi F, Stanton C, Evans S, Barnicot K, Sedgwick R, Personality disorder services in England: findings from a national survey. BJPsych Bull 2017; 41(5): 247–53. 29018548 10.1192/pb.bp.116.055251 PMC5623882

[2] Sheridan Rains L, Echave A, Rees J, Rachel Scott H, Lever- B, Broeckelmann E, Service user experiences of community services for complex emotional needs: a qualitative thematic synthesis. PLoS One 2021; 16(4): e0248316. 33914750 10.1371/journal.pone.0248316 PMC8084224

[3] Lohman MC, Whiteman KL, Yeomans FE, Cherico SA, Christ WR. Qualitative analysis of resources and barriers related to treatment of borderline personality disorder in the United States. Psychiatr Serv 2017; 68(2): 167–72. 27691382 10.1176/appi.ps.201600108 PMC5288272

[4] Borschmann R, Moran P. Crisis management in borderline personality disorder. Int J Soc Psychiatry 2011; 57(1): 18–20. 21252352 10.1177/0020764009106599

[5] Department of Health. Mental Health Crisis Care Concordat Improving Outcomes for People Experiencing Mental Health Crisis. Department of Health, 2014 (https://assets.publishing.service.gov.uk/government/uploads/system/uploads/attachment_data/file/281242/36353_Mental_Health_Crisis_accessible.pdf).

[6] Vandyk A, Bentz A, Bissonette S, Cater C. Why go to the emergency department? Perspectives from persons with borderline personality disorder. Int J Ment Health Nurs 2019; 28(3): 757–65. 30779279 10.1111/inm.12580

[7] Warrender D, Bain H, Murray I, Kennedy C. Perspectives of crisis intervention for people diagnosed with ‘borderline personality disorder’: an integrative review. J Psychiatr Ment Health Nurs 2021; 28(2): 208–36. 32367638 10.1111/jpm.12637

[8] Artis L, Smith JR. Emergency department staff attitudes toward people who self-harm: exploring the influences of norms and identity. Adv Emerg Nurs J 2013; 35(3): 259–69. 23899950 10.1097/TME.0b013e31829d202b

[9] Borschmann R, Trevillion K, Henderson RC, Rose D, Szmukler G, Moran P. Advance statements for borderline personality disorder: a qualitative study of future crisis treatment preferences. Psychiatr Serv 2014; 65(6): 802–7. 24585205 10.1176/appi.ps.201300303

[10] Helleman M, Goossens PJJ, Kaasenbrood A, van Achterberg T. Experiences of patients with borderline personality disorder with the brief admission intervention: a phenomenological study. Int J Ment Health Nurs 2014; 23(5): 442–50. 24890615 10.1111/inm.12074

[11] Borschmann R, Henderson C, Hogg J, Phillips R, Moran P. Crisis interventions for people with borderline personality disorder. Cochrane Database Syst Rev 2012; 6: CD009353. 10.1002/14651858.CD009353.pub2 22696385

[12] Chilman N, Morant N, Lloyd-Evans B, Wackett J, Johnson S. P30 #CrisisTeamFail: Twitter analysis to enrich understandings of mental health crisis services. BMJ Open 2019; 9(Suppl 1): A25.1–A25.

[13] Lawn S, Mcmahon J. Experiences of care by Australians with a diagnosis of borderline personality disorder. J Psychiatr Ment Health Nurs 2015; 22(7): 510–21. 26122817 10.1111/jpm.12226 PMC4755162

[14] Wood L, Alsawy S. Patient experiences of psychiatric inpatient care: a systematic review of qualitative evidence. J Psychiatr Intensive Care 2016; 12(1): 35–43.

[15] Royal College of Psychiatrists. Services for People Diagnosable with Personality Disorder. Royal College of Psychiatrists, 2020 (https://www.rcpsych.ac.uk/docs/default-source/improving-care/better-mh-policy/position-statements/ps01_20.pdf?sfvrsn=85af7fbc_2).

[16] National Institute for Mental Health in England. Breaking the Cycle of Rejection: The Personality Disorder Capabilities Framework. Department of Health, 2006 (http://personalitydisorder.org.uk/wp-content/uploads/2015/06/personalitydisorders-capabilities-framework.pdf).

[17] Sandelowski M, Docherty S, Emden C. Focus on qualitative methods qualitative metasynthesis: issues and techniques. Res Nurs Health 1997; 20(4): 365–71. 9256882 10.1002/(sici)1098-240x(199708)20:4<365::aid-nur9>3.0.co;2-e

[18] Moher D, Liberati A, Tetzlaff J, Altman DG. Preferred reporting items for systematic reviews and meta-analyses: the PRISMA statement. Int J Surg 2010; 8(5): 336–41. 20171303 10.1016/j.ijsu.2010.02.007

[19] Hannes K, Lockwood C, Pearson A. A comparative analysis of three online appraisal instruments’ ability to assess validity in qualitative research. Qual Health Res 2010; 20(12): 1736–43. 20671302 10.1177/1049732310378656

[20] Thomas J, Harden A. Methods for the thematic synthesis of qualitative research in systematic reviews. BMC Med Res Methodol 2008; 8: 45. 18616818 10.1186/1471-2288-8-45 PMC2478656

[21] Dunne E, Rogers B. ‘It's us that have to deal with it seven days a week’: carers and borderline personality disorder. Community Ment Health J 2013; 49(6): 643–8. 23054157 10.1007/s10597-012-9556-4

[22] Morris C, Smith I, Alwin N. Is contact with adult mental health services helpful for individuals with a diagnosable BPD? A study of service users views in the UK. J Ment Heal 2014; 23(5): 251–5. 10.3109/09638237.2014.951483 25222368

[23] Spence JM, Bergmans Y, Strike C, Links PS, Ball JS, Rhodes AE, Experiences of substance-using suicidal males who present frequently to the emergency department. CJEM 2008; 10(4): 339–46. 18652726 10.1017/s1481803500010344

[24] Barr KR, Jewell M, Townsend ML, Grenyer BFS. Living with personality disorder and seeking mental health treatment: patients and family members reflect on their experiences. Borderline Pers Disord Emot Dysregulation 2020; 7: 21. 10.1186/s40479-020-00136-4 PMC7487914 32944249

[25] Eckerström J, Flyckt L, Carlborg A, Jayaram-Lindström N, Perseius KI. Brief admission for patients with emotional instability and self-harm: a qualitative analysis of patients’ experiences during crisis. Int J Ment Health Nurs 2020; 29(5): 962–71. 32406168 10.1111/inm.12736

[26] Burke L, Kells M, Flynn D, Joyce M. Exploring staff perceptions of the utility of clinician connections when working with emotionally dysregulated clients. Borderline Pers Disord Emot Dysregulation 2019; 6: 12. 10.1186/s40479-019-0109-0 PMC6660965 31372226

[27] Commons Treloar AJ. A qualitative investigation of the clinician experience of working with borderline personality disorder. NZ J Psychol 2009; 38(2): 30–4.

[28] Liljedahl SI, Helleman M, Daukantaité D, Westrin Å, Westling S. A standardized crisis management model for self-harming and suicidal individuals with three or more diagnostic criteria of borderline personality disorder: the Brief Admission Skåne randomized controlled trial protocol (BASRCT). BMC Psychiatry 2017; 17: 220. 28619050 10.1186/s12888-017-1371-6 PMC5472925

[29] Borschmann R, Barrett B, Hellier JM, Byford S, Henderson C, Rose D, Joint crisis plans for people with borderline personality disorder: feasibility and outcomes in a randomised controlled trial. Br J Psychiatry 2013; 202(5): 357–64. 23637110 10.1192/bjp.bp.112.117762

[30] Veysey S. People with a borderline personality disorder diagnosis describe discriminatory experiences. Kotuitui 2014; 9(1): 20–35.

[31] Fallon P. Travelling through the system: the lived experience of people with borderline personality disorder in contact with psychiatric services. J Psychiatr Ment Health Nurs 2003; 10(4): 393–401. 12887630 10.1046/j.1365-2850.2003.00617.x

[32] Cree L, Brooks HL, Berzins K, Fraser C, Lovell K, Bee P. Carers’ experiences of involvement in care planning: a qualitative exploration of the facilitators and barriers to engagement with mental health services. BMC Psychiatry 2015; 15: 208. 26319602 10.1186/s12888-015-0590-y PMC4553006

[33] Sweeney A, Fahmy S, Nolan F, Morant N, Fox Z, Lloyd-Evans B, A mixed-methods study exploring therapeutic relationships and their association with service user satisfaction in acute psychiatric wards and crisis residential alternatives. Heal Serv Deliv Res 2014; 2(22): 1–106. 25642512

[34] Osborn DPJ, Lloyd-Evans B, Johnson S, Gilburt H, Byford S, Leese M, Residential alternatives to acute in-patient care in England: satisfaction, ward atmosphere and service user experiences. Br J Psychiatry 2010; 197(Suppl 53): 41–5. 10.1192/bjp.bp.110.081109 20679279

[35] Johnson S, Dalton-Locke C, Vera San Juan N, Foye U, Oram S, Papamichail A, Impact on mental health care and on mental health service users of the COVID-19 pandemic: a mixed methods survey of UK mental health care staff. Soc Psychiatry Psychiatr Epidemiol 2021; 56: 25–37. 10.1007/s00127-020-01927-4 PMC7453694 32857218

[36] Morant N, Lloyd-Evans B, Lamb D, Fullarton K, Brown E, Paterson B, Crisis resolution and home treatment: stakeholders’ views on critical ingredients and implementation in England. BMC Psychiatry 2017; 17: 254. 28716022 10.1186/s12888-017-1421-0 PMC5512942

[37] Bion WR. Learning from Experience. Heinemann Medical Books, 1962.

[38] Gibson R, Till A, Adshead G. Psychotherapeutic leadership and containment in psychiatry. BJPsych Adv 2019; 25(2): 133–41.

[39] Troup J, Taylor BL, Rains LS, Broeckelmann E, Russell J, Jeynes T, Clinician perspectives on what constitutes good practice in community services for people with Complex Emotional Needs: A qualitative thematic meta-synthesis. medRxiv [Preprint] 2020. Available from: https://www.medrxiv.org/content/10.1101/2020.12.15.20248267v1 [cited 1 Jun 2021]. 10.1371/journal.pone.0267787 PMC9070883 35511900

[40] Jennings P, Matheson-Monnet CB. Multi-agency mentoring pilot intervention for high intensity service users of emergency public services: the Isle of Wight Integrated Recovery Programme. J Criminol Res Policy Pract 2017; 3(2): 105–18.

[41] StopSIM Coalition. *StopSIM Coalition Consensus Statement relating to The High Intensity Network (HIN) and Serenity Integrating Mentoring (SIM)*. StopSIM, 2021 (https://stopsim.co.uk/2021/04/21/stopsim-coalition-consensus-statement/).

